# COGNITION PROFILES AMONG OLDER BLACK ADULTS, BY SCHOOL DESEGREGATION STATUS, EDUCATIONAL, OCCUPATION, INCOME

**DOI:** 10.1093/geroni/igae098.3040

**Published:** 2024-12-31

**Authors:** Collin Perryman, Roland Thorpe, Keith E Whitfield

**Affiliations:** Johns Hopkins University, Baltimore, Maryland, United States; Johns Hopkins University, Baltimore, Maryland, United States; University of Nevada Las Vegas, Las Vegas, Nevada, United States

## Abstract

**Objective:**

We sought to identify the subgroups regarding the school environment (desegregated or segregated), educational attainment, occupation type, income, and cognition among older Black adults.

**Methods:**

Data from older Black adults (n=602; age range: 48 to 95 years) from the Baltimore Study of Black Aging—Patterns of Cognitive Aging (BSBA-PCA) was used to explore the various cognitive function subgroups. We conducted a latent class analysis (LCA) to understand the cognitive domain subgroups. The continuous indicators were several cognitive domains (global cognition, reasoning, memory, immediate recall, working memory, language, and perceptual speed), and the categorical indicators were school type, educational attainment, occupation type, and income.

**Results:**

We identified subgroup for each cognitive domain. For global cognition (1) highest educational attainment, (2) untrained occupation, and (3) most segregated; working memory (1) high educational attainment, (2) segregated, and (3) low monthly income; reasoning (1) high educational attainment; (2) mostly desegregated, and (3) segregated; memory (1) highly educated, (2) lowest income, and (3) segregated; perceptual speed (1) highly educated, (2) lowest income, and (3) segregated. Given all the indicators, each subgroup exhibited an incremental increase in mean cognition scores.

**Conclusion:**

Our study provides insights to how cognition varies by school setting, educational attainment, occupation type, and income among older Black adults who attended desegregated or segregated schools in their early-life.

